# Novel approaches in cancer treatment: preclinical and clinical development of small non-coding RNA therapeutics

**DOI:** 10.1186/s13046-021-02193-1

**Published:** 2021-12-04

**Authors:** Rossana Cuciniello, Stefania Filosa, Stefania Crispi

**Affiliations:** 1 Institute of Biosciences and BioResources-UOS Naples CNR, via P. Castellino, 111-80131 Naples, Italy; 2 IRCCS Neuromed, Pozzilli, IS Italy

**Keywords:** Cancer therapy, RNA interference, microRNA (miRNA), Small interfering RNA (siRNA), Nanoparticles, sncRNAs therapeutics

## Abstract

Short or small interfering RNAs (siRNAs) and microRNA (miRNAs) are molecules similar in size and function able to inhibit gene expression based on their complementarity with mRNA sequences, inducing the degradation of the transcript or the inhibition of their translation.

siRNAs bind specifically to a single gene location by sequence complementarity and regulate gene expression by specifically targeting transcription units via posttranscriptional gene silencing. miRNAs can regulate the expression of different gene targets through their imperfect base pairing.

This process - known as RNA interference (RNAi) - modulates transcription in order to maintain a correct physiological environment, playing a role in almost the totality of the cellular pathways.

siRNAs have been evolutionary evolved for the protection of genome integrity in response to exogenous and invasive nucleic acids such as transgenes or transposons. Artificial siRNAs are widely used in molecular biology for transient silencing of genes of interest. This strategy allows to inhibit the expression of any target protein of known sequence and is currently used for the treatment of different human diseases including cancer.

Modifications and rearrangements in gene regions encoding for miRNAs have been found in cancer cells, and specific miRNA expression profiles characterize the developmental lineage and the differentiation state of the tumor. miRNAs with different expression patterns in tumors have been reported as oncogenes (oncomirs) or tumor-suppressors (anti-oncomirs). RNA modulation has become important in cancer research not only for development of early and easy diagnosis tools but also as a promising novel therapeutic approach.

Despite the emerging discoveries supporting the role of miRNAs in carcinogenesis and their and siRNAs possible use in therapy, a series of concerns regarding their development, delivery and side effects have arisen.

In this review we report the biology of miRNAs and siRNAs in relation to cancer summarizing the recent methods described to use them as novel therapeutic drugs and methods to specifically deliver them to cancer cells and overcome the limitations in the use of these molecules.

**Supplementary Information:**

The online version contains supplementary material available at 10.1186/s13046-021-02193-1.

## Background

Cancer is a complex genetic disease mainly due to dysregulation in the expression of genes involved in critical cellular pathways. Carcinogenesis can be due to the alteration of the expression of coding sequences such as oncogenes or oncosuppressors, but also to the misregulation of non-coding elements whose transcription generates non-coding RNAs [1].

Non-coding RNAs for a long time after discovery were thought to be non-functional molecules and they were considered as “transcriptional noise”. Subsequently, it was recognized that non-coding RNAs play a key role as transcriptional and translational regulators in different diseases including cancer [2]. This new biological role stimulated scientists in analyzing the possibility of translating their effectiveness in clinics, starting to consider them as novel drugs for the treatment of cancer and other diseases [3]. Non-coding RNAs are molecules classified according to their length: those up to 200 nucleotides long are called small non-coding RNAs (sncRNA), the others are known as long non-coding RNAs (lncRNAs) [4].

sncRNAs include microRNA (miRNAs), small interfering RNAs (siRNAs) and other small RNAs such as small nuclear RNAs (snRNAs), small nucleolar RNAs (snoRNA), Piwi-interacting RNAs (piRNAs) and transfer RNAs (tRNAs) [5].

miRNAs and siRNAs are the only sncRNAs that have been studied for therapeutics and their biology and application as anticancer will be described in this review thoroughly.

miRNAs and siRNAs are the sncRNAs widely distributed in both phylogenetic and physiological terms and are characterized by the double-stranded nature of their precursors. These sncRNAs are related in size, biogenesis and mechanism of action. miRNAs are small molecules implicated in the mechanism of RNA interference (RNAi) that performs a fine regulation of gene expression process by interfering with gene transcription thus affecting the fate of their target messenger RNAs (mRNA) that can be repressed or degraded. Similarly, siRNAs can knockdown the expression of target genes in a sequence-specific way by inducing mRNA degradation [6].

miRNAs were first described in the 1990s by Lee and colleagues that observed in *C. elegans* the formation of small RNAs produced by the transcription of lin-4 locus and characterized by an antisense complementarity to lin-14 gene [7]. Several years later these molecules were identified as miRNAs and since then, through cloning, sequencing and computational prediction, thousands of miRNAs were identified in different organisms. It is estimated that miRNA coding genes represent 1–5% of the mammalian genes [8].

Both miRNAs and siRNAs inhibit transcription by binding specific sequences of mRNA: miRNAs can recognize targets by perfect or imperfect complementarity; siRNAs inhibit transcription by creating a double strand molecule that binds only perfect complementary sequences on target mRNA [9]. Considering the ability of these molecules to hypothetically target the expression of any gene, starting from their discovery sncRNAs have been studied as possible tools to be used in translational and clinical approaches related in cancer and in many other diseases [10]. Although the similar activity of miRNAs and siRNAs, their application from bench to therapy are different since miRNAs are able to modulate simultaneously the expression of several different target genes, while siRNAs can specifically target a single gene at a time [11, 12].

Emerging therapeutic strategies based on the use of miRNAs and siRNAs are under development, with the purpose of making sncRNAs-based therapeutics a new and powerful tool to treat cancer [13, 14]. Several studies and clinical trials have been dedicated to the development of novel anticancer treatments miRNA and siRNA-based. miRNA-based therapeutics can determine miRNA inhibition or miRNA replacement. siRNA-based therapeutics by inhibiting the expression of a specific mRNAproduce a gene silencing effect [10, 15].

In this review we discuss the recent progress of small RNAs-based cancer therapeutics detailing the differences between using miRNAs and siRNAs. We also report recent advances in the field that will provide valuable progress to cancer therapeutics.

Specifically, we performed a detailed literature review updated to to October 2021, searching within the main public scientific databases (Supplemental Fig. 1).

### Small non coding RNA biogenesis and mechanism of action

RNA silencing is a gene regulatory system that can act either by suppressing transcription or degrading transcribed RNA. After the discovery of the first miRNA [8] it was described that exogenous double-stranded RNA (dsRNA) was able to silence gene expression through RNAi. RNAi was identified also in plants in which the silencing is concomitant with the presence of small RNAs 20–25 nucleotides long, perfectly matching to the sequence to be inactivated. Subsequent studies clarified that small RNA regulators are present in different plants and in animal species and that they can be divided into two categories: miRNAs that regulate endogenous genes and siRNAs that act to keep genome integrity against external insult of nucleic acids (viruses, transposons, transgenes) [16].

Despite their different mechanisms of action, miRNAs and siRNAs have similar physical and chemical properties, being both short RNA duplexes that target mRNAs and determine gene silencing.

miRNAs and siRNAs activity both depend upon Dicer and Argonaute (AGO), two proteins crucial for small RNA regulatory pathways: Dicer produces small RNAs from their double-stranded precursors, and AGO binds mature small RNAs letting them to exert gene silencing function [17, 18].

The modulation of endogenous or exogenous genes represents a powerful reprogrammable and tunable system to regulate gene expression.

**Fig. 1:**
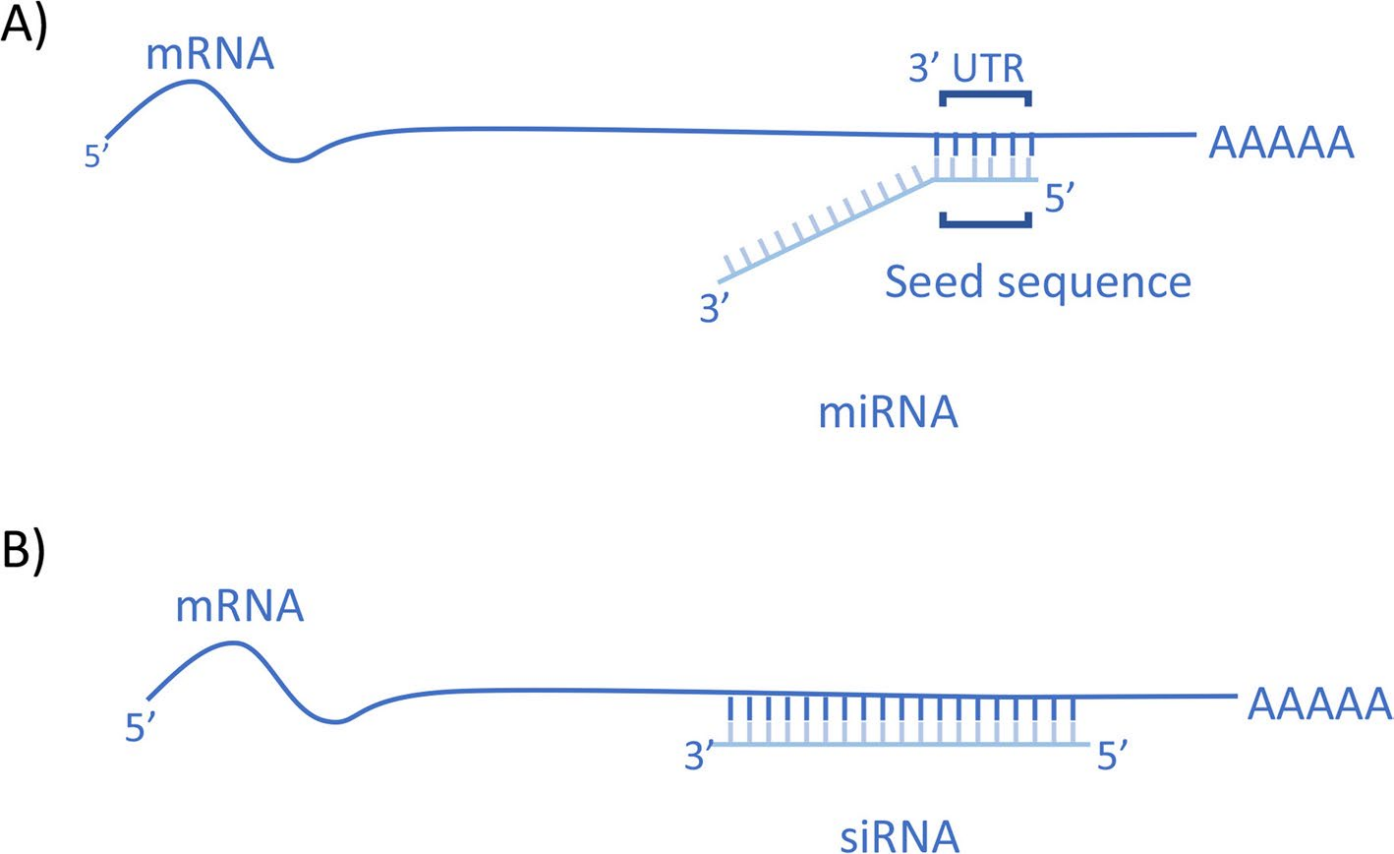
miRNA and siRNA structures. **A** miRNAs are characterized by a 7 nucleotide “seed sequence” that determines gene silencing by binding the 3′ UTR of mRNA target. **B** siRNAs bind fully complementary sequences on the mRNA target that is than degraded

Mature miRNAs are about 22 nucleotides long, but they are convergently transcribed from longer genes generally located in the introns of the pre-mRNA host genes [19, 20]. Mature miRNA contains at 5’end a 7 nucleotides sequence (seed sequence) that matches the 3′ UTR of mRNA target thus determining gene downregulation [21, 22] (Fig. 1).

**Fig. 2:**
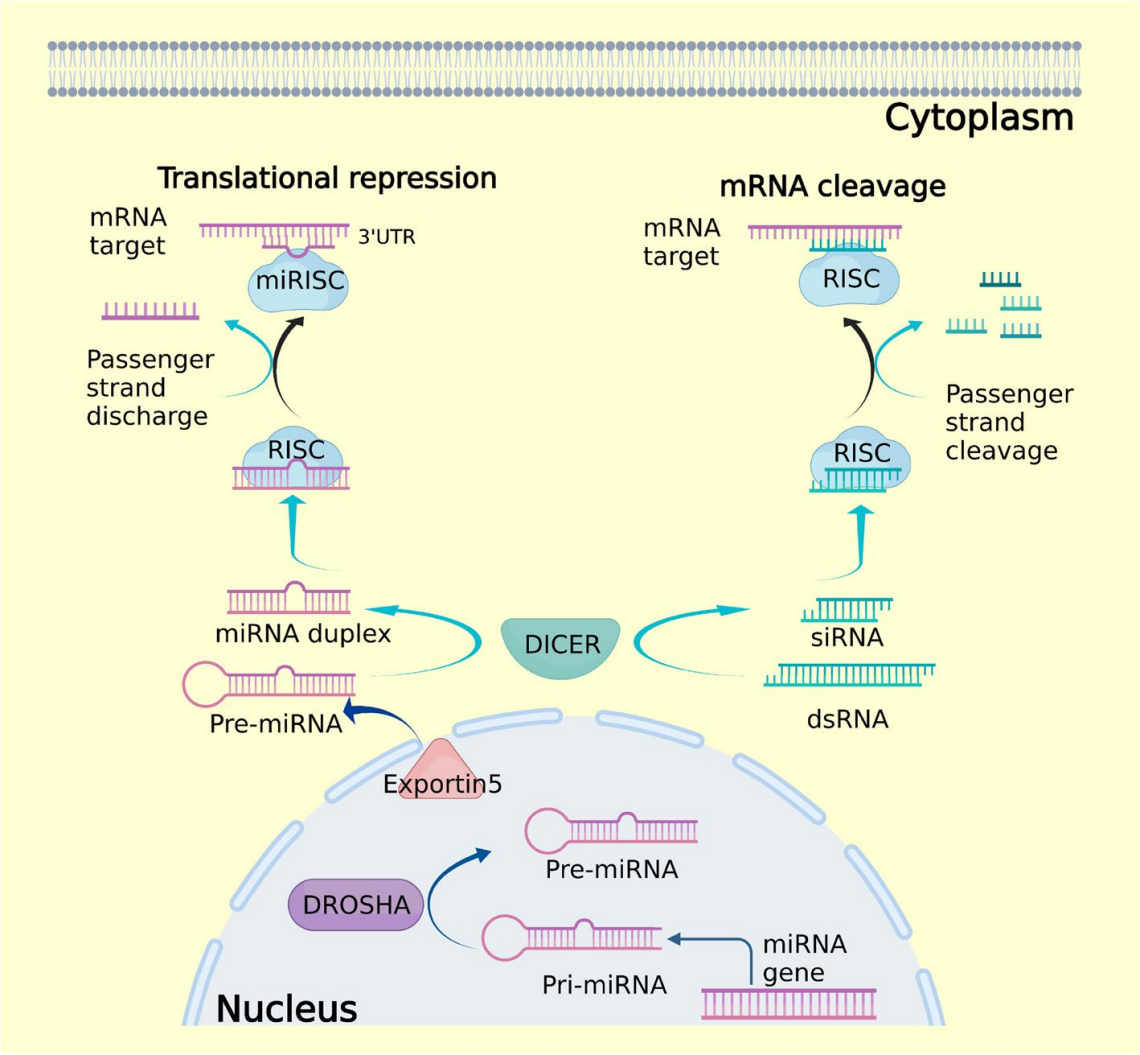
miRNA and siRNA biogenesis. In the nucleus the transcribed Pri-miRNA are converted by DROSHA in Pre-miRNA. Then they are translocated in the cytoplasm by Exportin 5. In the cytoplasm, DICER cleaves the Pre-miRNA in miRNA duplex and then the RISC complex selects the guide strand with consequent RNA target repression. Exogenous dsRNAs are converted in the cytoplasm in siRNA by DICER. Then siRNA is loaded in the RISC complex and, after the cleavage of the passenger strand, the guide strand determines mRNA degradation

miRNAs biogenesis takes place in the nucleus where they are transcribed by RNA Polymerase II as longer precursor (pri-miRNA) [23]. The pri-miRNA is converted into pre-miRNA [24, 25] and then it is translocated to the cytoplasm where it is shortened from a 70 nucleotides stem-loop structure to a 18–24 base pair double-strand RNA [26, 27]. Then two strands undergo different processes. The strand named “guide strand” is integrated in the RISC (RNA-induced silencing complex) AGO complex while the other one, the “passenger strand”, is discharged from the protein complex. Theguide strand mediates gene silencing on the target mRNA by translational repression or mRNA degradation [28, 29] (Fig. 2).

The stability of base pairing of the 2–4 nucleotides at the 5′ end of the duplex dictates which strand becomes the guide strandand the efficacy of the silencing is dictated by the nucleotides composition of the “seed sequence” and by the degree of complementarity to the 3′ UTR of the target mRNA [30, 31].

Depending on the degree of complementarity, the silencing can result in either translational inhibition or in accelerating the shortening of the poly(A) tail, thus determining a faster mRNA degradation.

The mRNAs translationally repressed by miRNA can accumulate in cytoplasmatic foci known as P-bodies (Processing-bodies) or GW-bodies (Glycine-Tryptophan bodies), twostructures containing proteins involved in mRNA decay [32].

siRNAs were first observed by transgene-induced silencing experiments in plants and it was thought that they only derived from exogenous sequences [33]. Subsequent functional studies led to detect that trans-acting siRNAs also resulted from genomic transcripts. Afterward different sources of endogenous siRNAs were identified, and it was evidenced that both exogenous and endogenous siRNAs are subjected to the same processing mechanism, with the endogenous ones having an obligate nuclear phase [34, 35].

siRNAs are generated from perfectly-paired dsRNAs ranging from 30 to over 100 nucleotides. They are produced in cells as response to external insults such as RNA viruses that, replicating themselves, allow the formation of dsRNA intermediates. siRNAs can also be generated from the transcription of overlapping genes, or by RNA-dependent RNA polymerases that generate complementary strands from single-stranded RNA templates. The dsRNAs precursors are processed in the cytoplasm by the RNAse III Dicer, which cleaves the longer precursor sequence in shorter molecules, a siRNA 21–23 nucleotides long with two nucleotides 3′ overhangs. The two strands of mature siRNAs are then processed by the RISC complex. In this case, and differently from miRNAs maturation, the passenger strand is the sense strand while the antisense strand always represents the guide strand. dsRNAs bind and activate the RISC complex, and then AGO cleaves the passenger strand retaining the guide strand. The assembled RISC-siRNA complex is now ready totarget specific mRNAs with binding occurring between fully complementary sequences (Fig. 2). Subsequently, the endonuclease Slicer in the RISC effector complex cleaves the mRNA sequence complementary to the siRNA guide. The mRNA is then degraded by exonucleases and thus silenced [36–39].

miRNAs can modulate gene expression inducing translational repression, mRNA deadenylation or decapping but they can also activate transcription or translation [40].

In most cases, miRNAs induce mRNA degradation or translational repression interacting with the 3′ UTR of target mRNAs [41]. In addition, also the binding to 5′ UTR or to the gene coding sequence induces gene silencing [42, 43]. In contrast, the interaction of miRNAs with promoter regions has been reported to activate transcription [44].

As reported before the recognition between miRNAs and mRNAs does not require perfect pairing. For this reason, a miRNA can recognize and down-regulate the expression of different mRNAs. Interestingly, an incomplete base pairing between miRNA and mRNA, does not activate AGO of the RISC complex, but miRNA can mediate silencing through translational repression, or by degradation, deadenylation, decapping or exonuclease action. Of course, a perfect pairing of miRNA with mRNA generates a double strand RNA recognized by AGO that leads to endonucleolytic cleavage of mRNA [45].

Unlike miRNAs, siRNAs only determine long-term silencing of the mRNAs encoding genes. siRNA by binding only to specific mRNA targets, generates a perfect pairing double strand RNA that is cleaved by AGO [46].

### Strategies for sncRNA-based gene targeting in therapeutics

The incidence of cancer is rapidly increasing thus resulting in a high economic and social impact [47]. Conventional cancer treatments, such as removal of cancer tissues and metastases with surgery as well as the use of chemotherapeutics or radiotherapy, improved overall survival but, nevertheless, they fail to prevent cancer recurrence and metastasis [48–50]. Furthermore, chemotherapy determines serious adverse effects such as systemic toxicity and increases the multiple drug resistance, all issues requiring the development of new more effective therapeutic strategies. Trying to improve cancer therapeutics, emerging strategies based on sncRNAs molecules are under development, with the purpose of making miRNA and siRNA-based drugs as new and powerful tools to cure cancer [14].

Undesired expression of mutated genes or overexpression of certain genes can be the origin of different diseases including cancer. The major limitation of chemical drugs is that they act only on specific proteins or enzymes. In contrast, miRNAs and siRNAs due to their ability to modulate gene expression are able to act also on “non-druggable” targets and have the huge potential to be considered as therapeutic agents. For example, gene dysregulation that characterizes cancer cells can be restored using miRNA replacement therapy or miRNA inhibitors, with the aim to restore their physiological expression.

#### miRNAs as therapeutic agents

Considering that the majority of human genes contain at least one miRNA consensus site, miRNAs could be used in different therapeutic applications [51]. Two main strategies can be used to manipulate gene expression through miRNAs, depending on whether miRNA should be re-introduced (mimic) or downregulated (inhibitor) to modulate the amount of mRNA target in the cell.

**Fig. 3:**
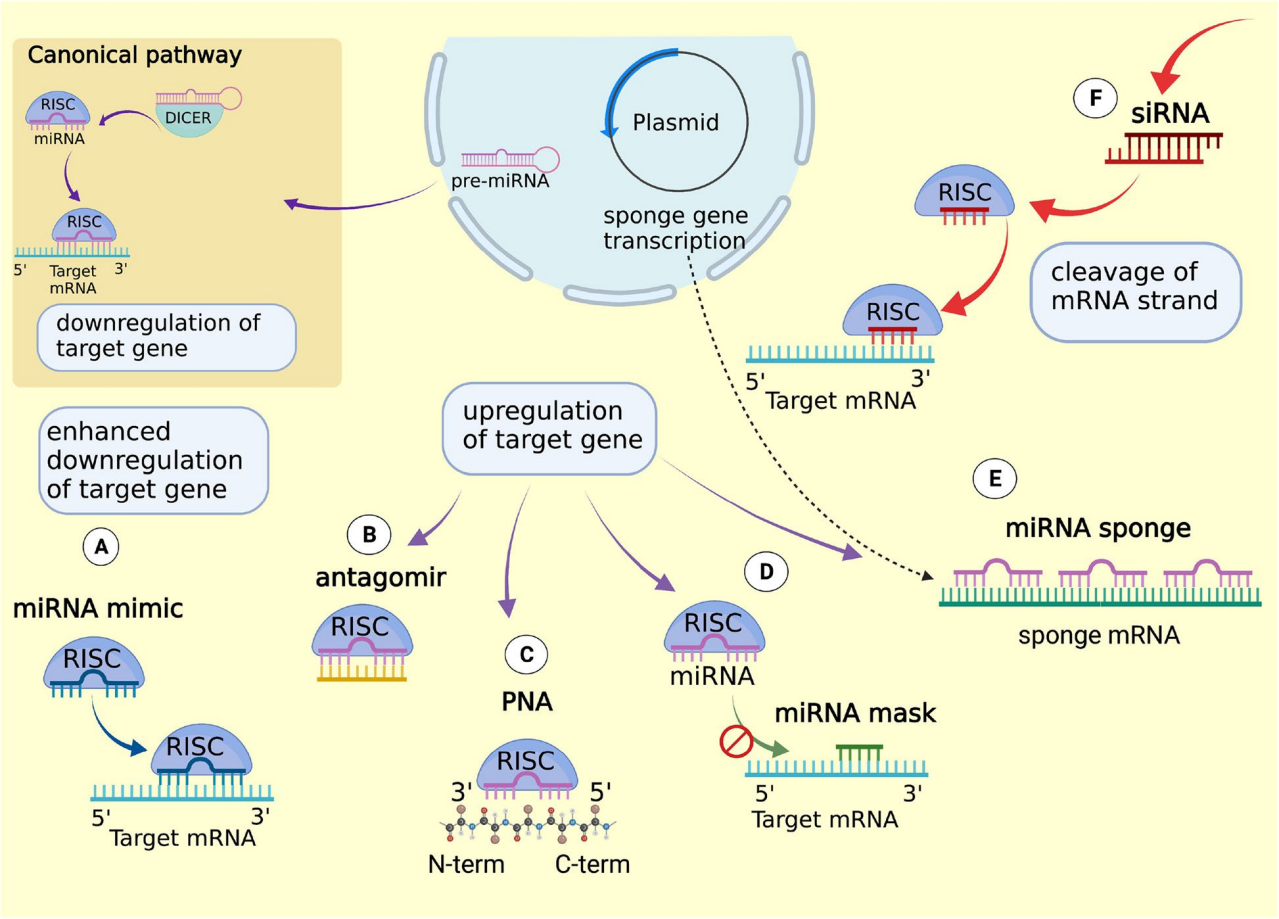
Schematic description of the strategies used to manipulate gene expression by sncRNAs: **A**) miRNA mimics; **B**) miRNA antagomirs; **C**) PNAs **D**) miRNA masks **E**) miRNA sponges and **F**) siRNAs

miRNA mimics are synthetic double-stranded oligonucleotides that overexpress the target miRNA sequences. They are specifically designed to achieve the same biological functions of the endogenous miRNAs resulting in downregulation of cancer cells. Ectopical expression of miRNAs could be done using vectors that overexpress the target miRNA or using miRNA mimics. In both methods, the reintroduced miRNA achieves the same biological functions of the endogenous miRNA, by silencing the target mRNA (Fig. 3 A). To exert this function, the synthetic miRNA should have a structure able to be loaded in the RISC complex, to operate as a guide strand and to recognize and then interfere with the mRNA targets. Thus, miRNA mimics design should be performed considering the chemical modification needed to improve their binding affinity, biostability and pharmacokinetic properties. For example, double-stranded molecules composed by both passenger and guide strand resulted in a better silencing effect, due to a more efficient loading of the RNA molecule in the RISC complex. Synthetic single stranded miRNAs are less suitable since they are rapidly degraded in biological fluids, and are characterized by a short half-life after administration [52–54].

As opposed to miRNA mimics, miRNA inhibitors are designed to specifically block the upregulated expression of miRNAs associated with cancer development. The most commonly used method for targeting specific miRNAs is based on the generation of antisense oligonucleotides (ASOs), or antagomir, that specifically bind the endogenous target miRNA inhibiting its function. ASOs are designed as a single-stranded structure complementary to the sense strand of the target miRNA (Fig. 3 B). They exert their function as competitive inhibitors [55, 56]. In this case it is possible to design molecules with higher affinity to miRNAs, by inserting specific modifications such as the insertion of several bicyclic RNA analogues that form a “locked” conformation thus ensuring a better hybridization in miRNAs targeting process. These molecules are known as Locked nucleic acids (LNAs) [57, 58]. A different structural modification, based on the development of heteroduplex oligonucleotides anti-miRNA, also shows improved suppression efficiency [59]. Another strategy to increase the targeting efficacy is based on the development of peptide nucleic acids (PNA) that specifically target full-length miRNAs by Watson-Crick recognition (Fig. 3 C). These structures are relatively stable since they are synthetic nucleic acid analogues that possess a neutral backbone and are resistant to enzymatic degradation [60]. Other powerful competitive miRNAs inhibitors are the miRNA sponges, that are transcribed from strong promoters and contain multiple tandem binding sites to target a miRNA of interest (Fig. 3 E). In the cells, miRNA sponges, competing with the native targets of miRNAs, allow increased expression of the mRNAs target. miRNA sponges are longer nucleic acids, expressed by transgenic vectors, that specifically inhibit miRNA with a complementary heptameric seed. Their peculiar structure allows inhibition of a single miRNA or of a miRNA family whose heptameric seed binding sequence is the same, allowing increased expression of all mRNAs that are modulated by inhibited miRNA. Using appropriate promoters, they should work in any kind of cell or transgenic model organism [61]. The main advantage of sponges is that the use of transgene overcomes the problems related to the oligonucleotides uptake [62]. However, a limitation of this approach is that sponges need to be administered in higher concentrations than ASOs, thus increasing the possible off targets.

The miRNA-mask is a different strategy able to silence the target gene by competing with the binding of miRNA that regulates that gene on the 3′UTR site. To be effective, the miRNA-mask should contain the recognition motif for a miRNA within the 3′UTR of the target gene and the sequences containing the miRNA-binding motif. The presence of this stretch is fundamental to achieve gene specificity of miRNA-mask action allowing miRNA-mask binding.

The binding of miRNA-mask to the binding site of miRNAs in 3′UTR of the target mRNA avoids the recognition of miRNA on the target mRNA and the consequent degradation by RISC complex, leading to a relief of translational repression without affecting miRNA levels (Fig. 3 D) [63].

#### siRNAs as therapeutic agents

siRNAs are double strands molecules containing 19–21 nucleotides with two nucleotide overhangs at the 3′ end, usually TT and UU, that allow them to be recognized by the RNAi machinery. In vitro studies have demonstrated that longer dsRNAs with 27 nucleotides are more efficient. This feature could be related to the fact that longer structures are more easily processed by Dicer allowing a better gene silencing activity [64]. It has also been demonstrated that the siRNAs for therapeutic applications should not be longer than 30 nucleotides to avoid an immune response through the activation of Interferon pathway [65].

siRNAs can exert RNA interference in a very specific manner by binding the target mRNAs with a complete sequence base pairing (Fig. 1). As for miRNAs, therapeutic effectiveness of siRNAs strongly depends on the molecule design.

A critical step is the selection of the mRNA sequence that needs to be recognized by siRNA. In fact, to achieve efficient and specific gene silencing it is crucial to select the optimal sequence, excluding potential off targets. Even if this problem can be addressed by using specific different prediction algorithms emerged in recent years, the siRNA efficacy should be experimentally validated [66, 67].

Another important issue to consider in siRNA therapeutics is that an incorrect orientation of the dsRNA could determine an incorrect selection of the guide strand determining the silencing of non-intended mRNA. As described before, the RNA interference proceeds, after the correct loading of the siRNA in the AGO/RISC complex, with the discard of the passenger strand and the use of the guide strand for the mRNA degradation (Fig. 3 F) [68]. The guide strand is selected according to the loading orientation of the molecule in the AGO protein and both strands could be selected as guide strand. Therefore, the dsRNA to be used as siRNAs needs to be designed to assure proper strand selection by the RISC complex. This can be carried out considering two parameters: the *asymmetry rule* and the *5′ nucleotide preference*.

The *asymmetry rule* is based on the finding that the thermostability of the two ends of the duplex contributes to the selection of the guide strand [30]. Consequently, the strand with a 5′ end that contains higher A/U content would be discarded due to its high stability. A correct loading of the dsRNA in the RNAi machinery will be achieved by designing a nucleotide sequence with lower stability that will be selected as guide strand.

The *5′ nucleotide preference* parameter is based on the specificity of AGO proteins toward a strand with a U or an A at the 5′ end that is selected as a guide strand. Thus, the passenger strand of the duplex should contain C and G to reduce the possibility of an incorrect selection of the guide strand [69].

Finally, also the nucleotide composition of siRNAs can affect the silencing efficacy. For example, the G/C content of siRNAs could affect the thermodynamic stability and the accessibility to the target site. siRNAs with very high content of G/C have been described to show reduced efficiency, while siRNAs with a content of G/C between 30 and 64% exert an efficient gene silencing effect [70, 71].

To manipulate gene expression through siRNAs it is essential to design a specific oligonucleotide sequence that perfectly matches with mRNA target to minimize the off-target effects.

Off-target effect can occur either in presence of high concentration of siRNA or when there is a*miRNA-like off-target effect* [72, 73]. Excessive siRNAs concentration, competing with the same protein machinery used by endogenous miRNAs, can cause off-target effect through the saturation of the RNAi machinery [74]. The *miRNA-like off-target effect* occurs when a siRNA induces a sequence-dependent regulation of unintended transcripts through the sequence complementarity to their 3′ UTRs. This can determine mRNA degradation mediated by partial sequence complementation thus resulting in a miRNA-like translational inhibition with a decrease of the protein level [75]. In particular, it happens if the design of the 5′ of the guide strand is not accurate or if the siRNA is totally or partially complementary to the 3′ UTR of the mRNA [72, 76, 77].

A common approach to resolve both events that determine off-targets is the use of the minimum possible concentration of siRNA, as the previous mechanisms are dependent from the concentration [74]. Another possibility is to use low concentrations of multiple siRNAs sequences targeting the same mRNA that, recognizing different off-targets, will reduce the risk of the off-target effect [69]. The *miRNA-likeoff targeteffect* could be also overcome by avoiding in the siRNA design the seed sequence of miRNAs or the 2–7 nucleotides of 5′ end, identifiable with the help of miRNA databases [72, 76, 77].

### sncRNA-based therapeutics in oncology

There is a growing interest of the pharma companies in identifying new molecules to be used as novel drugs against cancer. In this regard, sncRNAs have the potential to become a new class of drugs active in different diseases including cancer.

The recent advances in the identification of the molecular pathways involved in cancer onset and progression open new possibilities for cancer therapy. In this regard the identification of the sncRNA targets represent a valuable tool for gene silencing, enabling the suppression of oncogeneic factors. Recently, extensive analyses have been performed on the identification of novel strategies to specifically target oncogene and tumor suppressor genes. For example, many studies are reported for prostate cancer, the second most commonly occurring cancer and one of the leading causes of death in man. Recent studies led to identify sncRNAs able to inhibit target genes involved in this cancer pathogenesis [78]. Interestingly, most of thesesncRNAs have been tested on ongoing preclinicalstudies to unravel the efficacy of sncRNAs-based therapeutics (Supplemental Table 1).

Despite the large number of sncRNAs analysed as therapeutics, only 3 miRNAs (Table 1) and 10 siRNAs (Table 2) are currently in clinical trials as anticancer and they will be described below [79].

**Table 1:** miRNAs in cancer therapeutics

Target	Drug name	Cancer	Phase	ClinicalTrials.gov Identifier
Mir 16	TargomiRs	Malignant pleura mesothelioma, Non small-cell lung cancer	I	NCT02369198
Mir 155	MRG 106	Lymphomas, Leukemia	I	NCT02580552
Mir 34a	MRX34	Melanoma, Primary liver cancer; Hematologic malignancies	I Terminated	NCT01829971
Mir 34a	MRX34	Melanoma, Primary liver cancer; Hematologic malignancies	I/II Withdrawn	NCT02862145

**Table 2:** siRNAs in cancer therapeutics

Target	Drug name	Cancer	Phase	ClinicalTrials.gov Identifier
PKN3	Atu027	Carcinoma, Pancreatic Ductal	I	NCT00938574
PKN3	Atu027	Carcinoma, Pancreatic Ductal	I/II	NCT01808638
KRAS	siG12D LODER	Pancreatic ductal adenocarcinoma, pancreatic cancer	I	NCT01188785
KRAS	siG12D LODER	Pancreatic ductal adenocarcinoma, pancreatic cancer	II	NCT01676259
KrasG12D mutation	Mesenchymal stromal cells-derived exosomes with KRAS G12D siRNA	Pancreatic cancer	I	NCT03608631
PLK1	TKM-080301	Adrenal cortical carcinoma, neuroendocrine tumor, hepatocellular carcinoma	I/II	NCT01262235
PLK1	TKM-080301	Adrenal cortical carcinoma, neuroendocrine tumor, hepatocellular carcinoma	I	NCT01437007
PLK1	TKM-080301	Adrenal cortical carcinoma, neuroendocrine tumor, hepatocellular carcinoma	I/II	NCT02191878
AR V7 variant	SXL01	Metastatic castration-resistant prostate cancer (CRPC)	I	NCT02866916
EphA2	EPHARNA	Advanced Malignant Solid Neoplasm	I	NCT01591356
BCL2L12	NU-0129	Gliosarcoma, recurrent Glioblastoma	I	NCT03020017
VEGF and KSP	ALN-VSP02	Solid tumors	I	NCT01158079
RRM2	CALAA-01	Cancer, solid tumor	I Terminated	NCT00689065
MYC	DCR-MYC	Hepatocellular Carcinoma, Solid Tumors; Multiple Myeloma, or Lymphoma	I Terminated	NCT02314052
MYC	DCR-MYC	Hepatocellular Carcinoma, Solid Tumors; Multiple Myeloma, or Lymphoma	Ib/II Terminated	NCT02110563

### miRNAs in clinical trials

#### TargomiRs

One of the first studies demonstrating an involvement of miRNA dysregulation in cancer reported that the loss of the miR-15a/16–1 resulted in chronic lymphocytic leukemia (CLL). miR-15a/16–1 was the first miRNA discovered to function as tumor suppressor by directly targeting BCL2 gene that inhibits apoptosis and that is one of the most important oncogenes involved in lymphoma development [80]. Two different mice carrying miR-15/16 deletion have been generated and both lines developed lymphomas. Although these mice showed only mild upregulation of BCL2 expression, a robust upregulation of several predicted miR-15/16 targets such as CCND1, CCND2, and IGF1R1 has been observed [81].

To analyse the anticancer effect miR-15/16 ectopic expression it was developed miR-16 mimic (TargomiRs) that was tested in a phase I clinical trial (NCT02369198) in patients with Malignant pleural mesothelioma or advanced non-small cell lung cancer. Results provided data that confirmed the activity of TargomiRs as inhibitor of tumor growth indicating that restoring the level of miRNAs that act as oncosuppressor can represent a very attractive way to inhibit tumor growth. The phase II has been planned to compare the TargomiRs effects with those obtained in patients after the second and third cycle of chemotherapy [82].

#### MRG-106

miR-155 is an oncogenic miRNA overexpressed both in hematological and in solid tumors and it is known to be a “bridge between inflammation and cancer” [83]. miR-155 is used as a diagnostic tool to distinguish between benign and malignant inflammation in cutaneous T-cell lymphoma (CTCL) since it is overexpressed in the skin of CTCL patients. miR-155 is a transcriptional target of STAT5 and upregulation of STAT5/miR-155 pathway has been described to be involved in proliferation of malignant T cells and it is also known that the antisense miR-155 inhibits the process. For this reason, miR-155 has been considered as a putative target for therapy in CTCL [84]. In vivo trials have been performed using an oligonucleotide inhibitor of miR-155 (MRG-106), which was able to activate the specific miR-155 targets. Interestingly, MRG-106 was shown to have a significant pharmacodynamic activity in the preclinical model and a phase I clinical trial (NCT03837457) evaluated its safety, tolerability, pharmacokinetics and preliminary efficacy. The preliminary results showed that MRG-106 was well-tolerated and the efficacy in the recovery of cutaneous lesions prompted additional therapeutic analysis [85].

#### MRX34

miR-34a is a tumor suppressor that is lost or under-expressed in different tumor types. Retrospective clinical studies reported a negative correlation between survival and reduction of miR-34a expression [86]. miR-34a downregulated the expression of many oncogenes, including MET, MYC, BCL2, PD-L1. In vitro studies showed that reintroduction of a miR-34a mimetic in tumor cell lines reduced cell proliferation, migration and invasion. In vivo, preclinical studies in animal models showed that miR-34a inhibited primary tumor growth, blocked metastasis, and improved survival. The effectiveness of miR-34a mimetic (MRX34) was then confirmed in mouse models of hepatocellular carcinoma [87].

Prompted by encouraging preclinical data, two phase I trials (NCT01829971 and NCT02862145) have been initiated to evaluate MRX34 safety, pharmacokinetics and clinical activity. The NCT01829971 recruited adult patients suffering from refractory advanced solid tumors or hematologic malignancies, the NCT02862145 enrolled patients with melanoma. The results of the first trial evidenced the anticancer activity and confirmed that miR-34a mimetic was able to modulate the expression of its targets. Unfortunately, both studies have been stopped due to a severe immune-mediated response [88].

### siRNAs in clinical trials

#### Atu027

Protein kinase N3 (PKN3) is a downstream effector of the PI3K-signal transduction pathway. This pathway is implicated in the control of morphology and locomotion of endothelial and cancer cells. PKN3 has been considered a promising therapeutic target to inhibit metastasis formation. Indeed, gene silencing of PKN by siRNA (Atu027) in vascular and lymphatic endothelial cells has been reported to inhibit tumor growth and lymph node metastasis formation in mouse models [89].

A phase I clinical trial (NCT00938574) demonstrated that Atu027 is well tolerated and has antitumor activity [90]. A subsequent phase I/II study (NCT01808638) has been started to evaluate the safety and activity of Atu027 in combination with the standard chemotherapeutic gentamicin, as a new treatment strategy for advanced pancreatic cancer disease. This trial confirmed the efficacy of Atu027 and confirmed the importance of continuing the study with the aim to use this molecule as standard drug for the treatment of advanced pancreatic carcinoma [91].

#### siG12D LODER

KRAS is a member of the small GTPase superfamily and mutated KRAS is considered a hallmark of pancreatic cancer. Suppression of this oncogene by RNAi was reported to inhibit growth both in vitro and in vivo [92, 93].

Since most pancreatic ductal adenocarcinomas are caused by KRAS G12D mutation, it has been developed a biodegradable matrix to deliver KRAS siRNA-G12D (siG12D LODER). The ability of this drug to silence the upregulated mutated gene and determine apoptosis of cancer cells, slowing the tumor growth, has been assessed and confirmed in an orthotopic mouse model [94].

A phase I clinical trial (NCT01188785) demonstrated that siG12D LODER is well tolerated, safe and has potential efficacy against pancreatic ductal adenocarcinomas [95]. In addition, a phase II (NCT01676259) continues to evaluate the efficacy of siG12D LODER in combination with chemotherapy. The patients have been enrolled, but, to date, the results have not been reported yet [96].

#### Mesenchymal stromal cells-derived exosomes loaded with KRAS G12D siRNA

Another phase I trial (NCT03608631) to try silencing the oncogene KRAS G12S is recruiting patients with metastatic pancreatic cancer. The aim of the study is to identify the best dose with reduced side effects of Mesenchymal Stromal Cells-derived Exosomes loaded with KRAS G12D siRNA [97]. In particular, patients affected by pancreatic ductal adenocarcinoma that overexpress KrasG12D mutation will be recruited. The treatment will evaluate the efficacy of the silencing and other parameters such as disease control rate and the median overall survival. For this trial the last update has been reported in April 2021 and the estimated study completion date will be March 2022.

#### TKM-080301

Polo-like kinases (PLKs) is a family of proteins, composed of at least 5 members, that has an important role in maintenance of mitotic integrity. Among them, PLK1 is the kinase involved in the control of mitotic entry, centrosome maturation, bipolar spindle formation, cohesion dissociation, chromosome congression and segregation, and cytokinesis PLK is over expressed in many types of tumors and its expression has been correlated with poor diagnosis.

Knockdown of PLK1 expression, using siRNA, induced a reduction in cell proliferation in different hepatocellular carcinoma cell [97]. Therefore, PLK1 was used as target for cancer treatment. Three phase I/II different clinical trials have been registered (NCT01262235 NCT01437007 NCT02191878) and all of them will evaluate the safety, pharmacokinetics and preliminary anti-tumor activity of siRNA against PLK1 (TKM-080301). TKM-080301 showed a favorable toxicity profile and preliminary anti-tumor efficacy has been observed. However,the clinical trials did not demonstrate improved survival in patientsand did not support further evaluation as a single agent [98].

#### SXL01 (PROSTIRNA)

Prostate cancers are the second leading cause of cancer death in men in the world. Hormone- resistant disease is characterized by overexpression of Androgen receptor (AR). These tumors do not respond to hormonal ablation and additionally, this practice often results in more aggressive cancer relapse.

It has been demonstrated that knocking down the androgen receptor by siRNA leads to significant apoptotic cell death, inhibiting the Bcl-xL–mediated survival signal that acts downstream of androgen receptor-dependent survival pathway [99].

In vivo*,* the efficacy of androgen receptor silencing has been tested in mouse models of prostate cancer. The results obtained suggested that this strategy efficiently knocks down androgen receptors and could be considered a new therapeutic approach for these cancers [100].

A phase I clinical trial (NCT02866916) has been carried out in humans using a siRNA, called SXL01(PROSTIRNA), to prevent the synthesis of the androgen receptor. The main goal of this trial is to evaluate the safety, the tolerability and the therapeutic effects of SXL01 in patients suffering from castration-resistant prostate carcinomas. Results have not been published until now. Therefore, in January 2021 the recruitment was withdrawn and the study cancelled.

#### EPHARNA

EphA2 is a tyrosine kinases receptor member of a largest subfamily composed of 14 receptors and 8 ligands. EphA2 is overexpressed in different cancers including breast, endometrial, lung, ovarian, pancreatic and prostate, and its expression is always associated with adverse outcomes. EphA2 acts as an oncoprotein influencing cell proliferation, survival, migration, invasion, and angiogenesis; moreover, it has been reported in preclinical studies that its down-regulation reduces tumorigenicity [101, 102]. All these data suggest that EphA2 is an ideal therapeutic target.

In vitro and in vivo studies demonstrated that EPHARNA, the EphA2-siRNA, reduces tumor growth dramatically and acts as an anti-angiogenic. Moreover, analysis in Rhesus macaques demonstrated that EPHARNA is well tolerated at all tested doses [103].

EPHARNA is currently used in a phase I clinical trial (NCT01591356) to study, also in patients with advanced metastatic solid cancer, the safety, the maximal tolerated dose and to determine its efficacy on tumor growth. The trial is ongoing and no results have been posted yet [104].

#### Nu-0129

Bcl2L12 is a member of the Bcl2 family, containing a Bcl-2 homology domain 2 (BH2). It has been described to have anti-apoptotic properties, but this role remains controversial in different cancer types. Although the full-length mRNA transcript of Bcl2L12 is expressed in many tissues, its overexpression in most human glioblastomas has been associated with tumor cell progression and tumor cell resistance to apoptosis. Conversely, knockdown of Bcl2L12 both in astrocytes and glioma cell lines resulted in enhanced apoptosis [105].

It has been registered an early phase I clinical trial (NCT03020017) to evaluate, in patients affected by recurrent glioblastoma multiforme or gliosarcoma, the safety of a drug called NU-0129. This drug should be able to target Bcl2L12 and stop cancer cells from growing.

NU-0129 is based on spherical nucleic acids (SNAs), and in particular consists of gold nanoparticle cores covalently conjugated with radially oriented and densely packed siRNA Bcl2L12 oligonucleotides. This novel nanotherapeutic is able to cross the blood brain barrier and the nucleic acid component is able to target Bcl2L12 allowing the induction of apoptosis.

Preliminary results showed that NU-0129 was well tolerated in glioblastoma patients with no adverse effects. There was also evidence that it is able to cross the blood brain barrier. Bcl2L12 expression and apoptotic markers analysis are pending [106].

#### ALN-VSP02

ALN-VSP02 is a RNAi-based therapeutic targeting the expression of vascular endothelial growth factor (VEGF)-A and kinesin spindle protein (KSP). VEGF-A is an angiogenic factor that promotes tumor-associated angiogenesis, inducing proliferation and migration of vascular endothelial cells. KSP plays an essential role in mitosis mediating centrosome separation, assembly and maintenance of mitotic spindle. Inhibition of KSR results in mitotic arrest and, ultimately, in apoptosis. Both VEGF-A and KSR are overexpressed in different types of tumors and different inhibitors of both proteins are under study as novel anticancer therapeutics.

In a phase I clinical trial (NCT00882180) the safety, tolerability, pharmacokinetics, and pharmacodynamics and the ability to reduce expression of VEGF-A and KSR of ALN-VSP02 was analysed in patients with advanced solid tumors with liver involvement. The results of the study showed that ALN-VSP02 is well tolerated, that the drug was delivered to the tumor and that VEGF mRNA was downregulated in a liver and extra hepatic metastasis. An extension of this phase I study (NCT01158079) has been started to analyse over the time the therapeutic efficacy of ALN-VSP02in patients who had clinical benefit as stable disease or better. The next phase II study will enrol patients treated with the established active dosage [107].

#### CALAA-01

Ribonucleotide reductase (RNR) is a ubiquitous rate-limiting enzyme that catalyzes de novo formation of deoxyribonucleotides. It is required for DNA synthesis and repair, and for maintaining a balanced dNTP pool. RNR is a tetrameric protein formed by two kinds of subunits: the large ribonucleotide reductase subunit M1 and two different small subunits RRM2 and RRM2B. High expression of RRM2 is common in cancers including melanoma where it influences survival, proliferation, apoptosis, and chemoresistance [108]. For this reason, RRM2 is a cancer therapeutic target.

CALAA-01 is a siRNA-based therapeutic interfering RRM2 that is able to recognize tumor cells expressing transferrin receptors. In phase I clinical trial (NCT00689065) CALAA-01 was well tolerated during the initial dose escalation. Furthermore, tumor biopsy from patients with metastatic melanoma showed that nanoparticles were localized in the tumor cells, but not in the surrounding normal epidermis and that RRM2 mRNA and protein levels were reduced [109].

#### DCR-MYC

c-Myc is a multifunctional transcription factor considered to be a “master regulator” of cellular metabolism and proliferation. Due to its important role as “primary oncoprotein” regulating many aspects of tumorigenesis, it represents a unique opportunity to develop novel cancer therapies [110].

DCR-MYC is a siRNA that specifically targets the oncogene MYC.

In a phase I clinical trial (NCT02110563) the safety in a dose-escalation study, the pharmacokinetics, the pharmacodynamics and clinical activity of DCR-MYC were analyzed in patients with advanced solid tumors, multiple myeloma or lymphoma in advanced solid tumors and hematological malignancies. The study demonstrated that DCR-MYC is well tolerated and shows promising initial clinical and metabolic responses [111]. In a phase Ib/II clinical trial (NCT02314052) the safety and tolerability ability to inhibit MYC of DCR-MYC were evaluated in patients with advanced hepatocellular carcinoma. Although the result of the phase I study supported the validation of MYC as a therapeutic target, both studies have been “Terminated” due to sponsor decision.

### siRNA -based cancer immunotherapy

Recent studies describe the use of specific siRNAs to develop specific cancer vaccines. These methods are based on the ability of siRNAs to produce a large number of antigens thus triggering a strong and specific immune response. In addition, cancer tumor antigenic proteins expressed in the cytoplasm of antigen presenting cells are able to induce a cytotoxic T cell response. This process involves the formation of a protein complex between MHC class I and peptide epitopes [112].

Ongoing cancer clinical trials based on siRNAs immunotherapy have been integrated with cell-based immunotherapy. In fact, in this way the immunogenicity of immune cells such as dendritic cells or T cells can be restored or enhanced. These immune cells are transfected ex-vivo with tumor-associated antigen encoding siRNAs through electroporation or nanoparticle approaches. Then the transfected cells are re-infused into the patient where they determine cancer cells death.

Different therapies developed in this way and that are undergoing phase I/II clinical trials are based on the use of siRNAs transfected dendritic cells [113].

Three siRNA-based immunotherapies are ongoing in clinical trial:

#### PSCT19

A phase I/II study (NCT02528682) used for hematological malignancies is based on siRNA immunotherapy by silencing programmed death-protein 1 (PD-1) and its ligands 1 and 2 (PD-L1/2). Their inhibition enhances T cell immune responses against tumor cells [114]. This method has been used also for solid tumor treatment.

#### iPsiRNA

A phase I clinical trial (NCT00672542) based on siRNA targeting immunoproteasome (iPsiRNA) subunits has been started to treat metastatic melanoma. The therapy is based on the different composition of the intracellular proteasomes in normal cells and in cancer cells. In fact, after exposure to inflammatory mediators normal cells change the proteasome from constitutive proteasome to immunoproteasome while cancer cells fail to express immunoproteasome. By modulating the proteasome of the mature dendritic cells, it is possible to stimulate a specific T cell response against melanoma cells. In this study, dendritic cells transfected with RNA encoding melanoma tumor-associated antigen stimulating a T cell response will be appropriately directed against melanoma cells [115].

#### APN401

A recent study based on the inhibition of Casitas-B-lineage lymphoma protein-b, an intracellular checkpoint limiting lymphocyte activation, has been initiated to treat different solid tumors. Previous studies in mouse models reported that the inhibition of this protein enhances the antitumor activity mediated by T cell and natural killer cell. APN401 is autologous cellular therapy consisting of peripheral blood mononuclear cells silenced for Casitas-B-lineage lymphoma protein-b. A first phase I clinical trial (NCT02166255) used a single intravenous infusion suspension of APN401 to treat patients with solid tumors; the results showed that APN401 increased cytokine production [116] and supported a second phase I clinical trial (NCT03087591) in which the effect of multiple infusions is tested [117].

### Delivery strategies for miRNA and siRNA therapeutics

The availability of small non-coding RNAs as cancer therapeutics represents a novel strategy to overcome pharmacological barriers involved in therapeutic resistance. The advantage in using sncRNAs in cancer therapy is that these molecules, affecting multiple signalling pathways, result more effectively in the majority of standard chemotherapeutics that usually target a single gene.

The major limitation in sncRNAs-based therapeutics is due to the difficulty in tuning their expression in cancer cells and also to the efficacy of their delivery into cells. Both these aspects should be taken into account when it is needed to translate to the clinic the use of sncRNAs molecules.

**Fig. 4:**
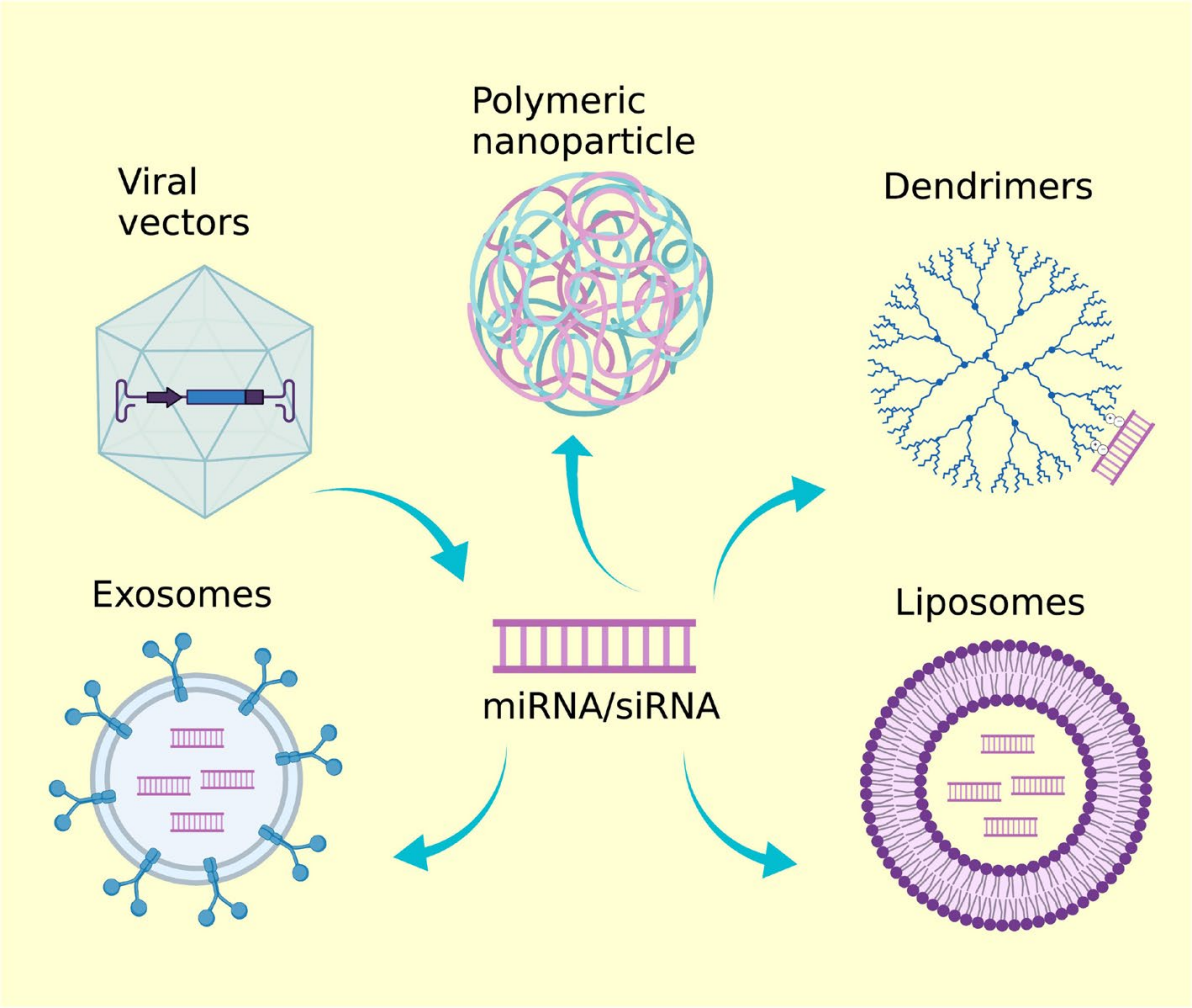
Structure of the delivery systems used for sncRNAs-based therapies

To be efficient a delivery system must guarantee that miRNAs and siRNAs reach their site of action, and avoid their degradation by nucleases present in biological fluids. Since miRNAs and siRNAs show similar chemical properties and exploit the same protein machinery to exert the gene silencing effect, similar delivery technologies can be developed for both molecules (Fig. 4).

As detailed above, the overexpression of a specific target miRNA can be carried out by using expression vectors or miRNA mimics and gene silencing can be realized by designing specific siRNAs.

miRNAs and siRNAs after entering into cells are converted into active, single-stranded molecules, but different limitations can result in poor effectiveness. For instance, the loss of RNA backbone modifications can prevent synthetic sncRNAs to reach their target location; they also can be degraded by nucleases present in the biological systems or can be poorly uptaken through cell membranes. To overcome these difficulties chemical modifications specific for each target tissue have been designed to optimize RNA oligonucleotide stability [118].

miRNAs and siRNAs both are characterized by low cellular uptake and can be easily degraded by nuclease-mediated degradation. Therefore, to have efficient delivery into target cells they should be delivered using proper carrier systems. Different delivery systems have been developed based on the use of viral systems, polymers, liposomes, and nanoparticles. In addition, the use of either lipids or exosomes greatly increases the uptake of synthetic sncRNAs.

The use of viral vectors to deliver sncRNAs is one of the first delivery systems developed. It is based on the use of viral vectors that show high transduction efficiency. In fact, RNA-encoding viruses are efficiently transferred in the nucleus of mammalian cells by adeno-associated viruses, adenoviruses and lentiviruses determining gene silencing [119]. Viral vectors have the advantage to efficiently deliver RNAs in vivo and allow stable gene silencing. For example, a vector-mediated RNAi -inducing gene cassette determined stable gene silencing thanks to the stable insertion of the vector into the host genome [120].

In addition, gene silencing can be tumor-targeted by modifying the capsid structures that regulate viral cell tropism thus driving viral vectors in specific cell types [121]. In the past years, viral vector-miRNA delivery has been used for the treatment of many diseases including cancer. For example, in a recent study an adeno-associated viral vector was used to restore the expression of *miR-122a* in a murine model of hepatocellular carcinoma using a virus with a strong liver tropism [122].

However, viral vectors have different limitations and adverse effects that should be solved before they can be used routinely in humans. In fact, high transgene expression can affect cell viability and sncRNA overexpression can result in cellular toxicity, off-target effects as well as in the induction of the immune interferon pathway. In addition, the recurrence of non-specific gene silencing and off-targeting issues makes it difficult to use them in clinical applications. Finally, to be used in therapy, viruses must be genetically engineered to remove their virulence and to alter their natural tropism toward specific cell types by modifying proteins of the viral capsid.

Despite the modifications that can be done on viral vectors, other serious safety concerns such as the risk of insertional mutagenesis in case of lentiviruses [123] and high immunogenicity in case of adenoviruses [124] stimulated scientists to develop non-viral vectors for siRNA and miRNA delivery with a better safety profile.

Among non-viral systems, polymer-based and lipid-based systems represent different and very flexible tools for RNA delivery. These systems can be modified in different ways to improve serum stability, half-life, and they can be used for site-specific delivery by using targeting ligands during formulation preparation. In addition, polymers and lipids have the advantage of being positively charged and thus they can associate to the negatively charged RNA [122, 125].

Among polymers, the cationic PEI (polyethylenimine) has been widely investigated for many years for the high efficiency delivery in vivo [126]*.* PEI acts as coating providing a net cationic charge while forming polyplexes with miRNA and siRNA, thus protecting them from degradation mediated by serum enzymes. It also enhances the introduction in the cell through the interaction with anionic cell membrane polysaccharides [127, 128]. Nanosized polyplexes are easy to prepare and endocytosis can facilitate their cellular uptake. The limitation of the use of this delivery system is represented by the possible interaction of the coating with serum proteins. In addition, due to its high toxicity PEI has been started to be used in clinics only after the development of specific modifications [126].

Polyphosphazenes are other synthetic polymers showing high biocompatibility and chemical flexibility that can be modified to achieve targeted release to the site of action. Different studies report their use to deliver sncRNAs in therapeutics [129].

Other natural cationic polymers such as PLGA (Poly (lactic-co-glycolic acid)) and cyclodextrins are considered as safe sncRNAs delivery systems.

PLGA is a synthetic biodegradable polymer-FDA approved, that has lower or absent toxicity. The advantage of PLGA is that with this polymer it is possible to modulate the drug release by changing its molecular weight and composition.

Cyclodextrins, cyclic oligomers of glucose, are other polymers widely accepted for human pharmaceutical formulations due to low toxicity, high stability and lack of immune stimulation.

Both PLGA and cyclodextrins have the advantage that they can be used to form nanoparticles in which sncRNAs can be loaded [130, 131].

Other attractive polymers used are the dendrimers, multi-branched polymers that contain a core surrounded by repeated iterations – the branches. Branches can have functional groups which can be modified for ligand attachment. Their structure allows them to complex many small RNAs molecules. Dendrimers can overcome intra- and extracellular barriers and have been used in different therapeutic fields [132, 133].

Unlike polymers, the use of lipids in vivo does not represent a good choice system due to toxicity, nonspecific uptake, and to the burst of inflammatory and immune responses. Often lipids nanoparticles are liposomes combined to PEG. Other strategies are based on the use of liposomes and protamine containing small RNA complexed to hyaluronic acid [134].

An evolution of cationic lipids used for sncRNAs delivery is represented by SNALPs (Stable nucleic acid lipid particles). These nanoparticles are characterized by the presence of an ionizable amino lipid into the lipid vesicles that facilitate the encapsulation of sncRNA with increase of serum stability. SNALPs have been used for the delivery of siRNA and miRNAs in different tumors and some SNALP-based formulations currently in clinical trials [135].

More recently, a combined system made of polymers and lipids has been developed and used for in vivo delivery of small RNAs. This system - named lipolyplexes - combines the advantageous characteristics of both molecules [136]. For example, Patisiran is a lipolyplexes clinically approved encapsulating siRNA directed against transthyretin mRNA. This delivery system after entering into the cell allows siRNA diffusion in the cytosol thanks to interactions between cationic lipids of the nanoparticles and anionic lipids of the endosomal membrane [137].

Lipoplexes are the most used delivery vehicles for sncRNA molecules and represent a promising delivery system since it is possible to modulate transfection efficiency and toxicity by changing the structure composition and /or the properties of the final formulation [138–140].

The commercial lipid-based systems for in vitro transfection are frequently used to deliver noncoding RNAs in cells with high efficiency, and in vivo performance has been recently improved. Thanks to the structural optimization developed in the last years, this delivery system has become highly efficient in capacity and in targeting specific cells. Interestingly, some lipoplexes formulations are currently investigated in clinical trials (ClinicalTrials.gov) for cancer treatment (Atu027, EphA2, DCR-MYC, TKM-08030).

A recent growing interest is related to the use of exosomes as sncRNAs delivery system. Exosomes can be viewed as the most challenging small RNA delivery systems since they can be efficient in drug delivery and can be produced at large-scale. Exosomes can be considered as lipid-based nanocarriers [141] being small membrane vesicles that are generated from cells. Discovered thirty years ago as components of the cellular waste disposal mechanism, exosomes are known to play a key role in the cellular signaling of cancer cells. Their formation lead to the production of vehicles charged with molecules coming from their cell of origin [142, 143].

Exosomes, being natural carriers of nucleic acids and proteins, represent the best candidate to deliver miRNAs and siRNAs for therapeutic purposes. In fact, due to their biological origin they are biocompatible and lack toxicity. Moreover, their small size allows them to easily encapsulate small RNA molecules through traditional transfection methods. In addition, due to their cellular nature exosomes escape phagocytic degradation and are naturally stable. Moreover, exosomes can cross the blood-brain barrier, thus can be useful also to develop brain cancer therapeutics [144].

The advantage in using exosomes as delivery systems is that they can be used for large-scale production with highly reproducible results. Finally, with exosomes it is possible to improve target specificity through genetic modification of their surface ligands.

Recent studies reported the efficacy of exosome-mediated delivery of siRNAs in different cancers such as breast cancer [145], leukemia [146] and glioma [147].

Finally, the relationship between exosomal miRNA deregulation and cancer can be used to develop targeted exosome-based miRNAs delivery. In fact, cancer exosomes have the ability to convert specific pre-miRNAs to mature-miRNAs, thus enriching them. The identification of miRNA exosome profile for a specific cancer allows the design of specific therapeutic exosomes to deliver the specific sncRNAs.

The development of delivery systems based on exosomes represents a very promising strategy to use these molecules in cancer therapeutics.

## Conclusions

Cancer is often a complex and multifactorial disease that nowadays still presents a lot of limitations in its treatment. Most of the therapies actually used to treat cancer are effective but present many side effects and their function can easily be reduced by multi-drug resistance. RNAi therapeutics are a very interesting and challenging possibility to treat cancer acting on the primary cause of its genesis, due to their possibility to target the mRNA expression of genes that are primarily involved in carcinogenesis. A lot of pre-clinical studies have demonstrated that miRNA and siRNA could be useful in diagnosing and treating cancer, but unfortunately most of the clinical trials involving them are still in phase I or II. A series of limitations have to be overcome to let these therapeutics be fully functional to treat this disease that still affects a lot of people worldwide. Despite all the limitations that slow down the approval of sncRNA-based therapeutics for cancer treatment, many studies have highlighted their functional use in this field. Specifically, miRNAs have been shown to be specific and sensible diagnostic and prognostic markers, easy to be dosed due to their presence in body fluids, while siRNAs are more specific molecules that can potentially inhibit the expression of any gene involved in cancer, without the limitations that characterize miRNAs. Since their discovery, miRNAs and siRNAs have become an important object of interest, and are still fascinating researchers from all medical fields. To date two sncRNAs–based therapeutics have been approved in clinics: ONPATTRO® (Patisiran) for treatment of polyneuropathy caused by hereditary transthyretin-mediated amyloidosis [148] and GIVLAARITM® (Givosiran) for the treatment of acute hepatic porphyria [149]. The increasing number of clinical trials with sncRNAs in cancer will allow to develop novel and efficient therapeutics approaches.

In clinical oncology the identification of potential prognostic factors –patient-related characteristics -is essential to assess a valuable prognosis, often independent from the treatment. A meta-analysis can be useful to identify predictive factors, thus allowing clinicians to select patient subgroups that possibly will benefit more from a specific treatment. To this aim the application of systematic meta-analysis will be important to evaluate the quality of a trial, and to quantify the overall treatment efficacy.

The importance of meta-analysis is that results are applicable to a broad spectrum of topics, including biomarkers, genetic factors, diagnosis, and treatment.

Meta-analysis application in clinical trials of sncRNAs therapeutics can be extended to other important areas, such as prognostic models, which are very important in oncology and should include individual studies. In addition, prognostic factors can be evaluated combining meta-analysis data and the data availablein real-world database [150]. The integration of these data might improve the construction of prognostic models and used to assess the efficacy and chose the better sncRNAs -based anticancer therapy.

Noncoding RNA therapeutics by modulating gene expression can act on several targets usually not reached by traditional chemical drugs that block the activity of specific proteins or enzymes. For this reason, they are a promising possibility to overcome the inability and the side effects related to the use of chemotherapeutics.

## Supplementary Information

**Additional file 1: Supplemental Figure 1**. Flow diagram summarizing how the study was conducted. A) Literature search was performed using PubMed and Web of Science databases using as keywords: small non coding RNAs, microRNAs, small interfering RNAs and cancer. Research reports, review articles and articles published up to October 2021 were evaluated. B) Clinical trials search was performed using the “Clinicaltrials.gov” database insertingthe same keywords as in A). Only the studies using miRNAs or siRNAs as drug were selected. Clinical trials in which miRNAs or siRNAs were evaluated as diagnostic or predictive tests were excluded.

**Additional file 2: Supplemental Table 1**. sncRNAs in prostate cancer preclinical trials.

## Data Availability

Not applicable.

## Abbreviations

AGO: Argonaute
Antagomir: antisense oligonucleotides
Anti-oncomirs: miRNA tumor-suppressors
AR: Androgen receptor
ASOs: antisense oligonucleotides
BH2: Bcl-2 homology domain 2
CLL: chronic lymphocytic leukemia
DGCR8: DiGeorge syndrome critical region 8
DOPC: 1,2-dioleoyl-sn-glycero-3-phosphatidylcholine
DsiRNA: Dicer-substrate small interfering RNA
dsRNA: double-stranded RNA
DUF283: Domain of unknown function
GW-bodies: Glycine-Tryptophan bodies
KSP: kinesin spindle protein
LNAs: Locked nucleic acids
lncRNAs: long non-coding RNAs
Mimic: miRNA re-introduced in the cell
miRNAs: microRNA
PNA: Peptide nucleic acids
PD-1: Programmed death-protein 1
PD-L1/2: Programmed death-protein ligands 1 and 2
PEI: polyethylenimine
piRNAs: Piwi-interacting RNAs
PIWI: P-element Induced Wimpy
PKN3: Protein kinase N3
PLGA: Poly lactic-co-glycolic acid
PLKs: Polo-like kinases
RISC: RNA-induced silencing complex
RNAi: RNA interference
RNR: Ribonucleotide reductase
RRM2: Ribonucleoside reductase M2
SNALPs: Stable nucleic acid lipid particles
SNAs: spherical nucleic acids
UTR: untranslated region
VEGF-A: vascular endothelial growth factor-A
