# Correction to: Novel approaches in cancer treatment: preclinical and clinical development of small non-coding RNA therapeutics

**DOI:** 10.1186/s13046-021-02228-7

**Published:** 2021-12-31

**Authors:** Rossana Cuciniello, Stefania Filosa, Stefania Crispi

**Affiliations:** 1 Institute of Biosciences and BioResources-UOS Naples CNR, via P. Castellino, 111-80131 Naples, Italy; 2 IRCCS Neuromed, Pozzilli, IS Italy

**Correction to: J Exp Clin Cancer Res 40, 383 (2021)**

**https://doi.org/10.1186/s13046-021-02193-1**

Following publication of the original article [1], it was noticed that Stefania Crispi was not indicated as corresponding author while her email address was listed correctly. This has now been acknowledged and corrected in this erratum. The original article has been corrected.
