# Supplementary figures and images for: Novel approaches in cancer treatment: preclinical and clinical development of small non-coding RNA therapeutics
Source: J Exp Clin Cancer Res. 2021 Dec 4;40:383. doi: 10.1186/s13046-021-02193-1 (PMC8642961; doi:10.1186/s13046-021-02193-1)

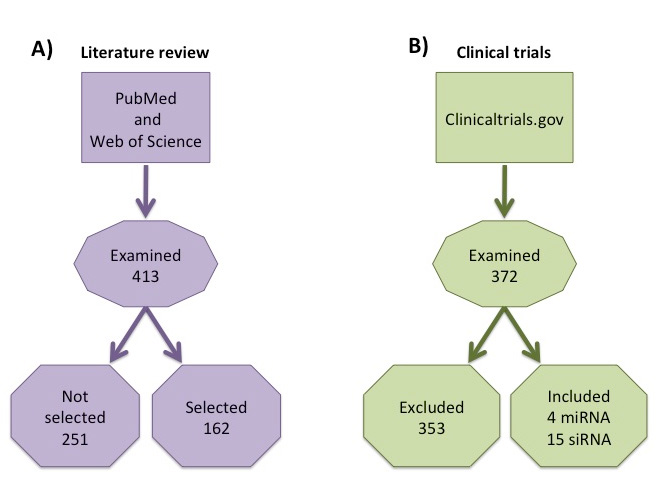

Supplement: Supplementary file 1 — Additional file 1: Supplemental Figure 1. Flow diagram summarizing how the study was conducted. A) Literature search was performed using PubMed and Web of Science databases using as keywords: small non coding RNAs, microRNAs, small interfering RNAs and cancer. Research reports, review articles and articles published up to October 2021 were evaluated. B) Clinical trials search was performed using the “Clinicaltrials.gov” database insertingthe same keywords as in A). Only the studies using miRNAs or siRNAs as drug were selected. Clinical trials in which miRNAs or siRNAs were evaluated as diagnostic or predictive tests were excluded. [file 13046_2021_2193_MOESM1_ESM.jpg]
