# Supplementary material for: Novel approaches in cancer treatment: preclinical and clinical development of small non-coding RNA therapeutics
Source: J Exp Clin Cancer Res. 2021 Dec 4;40:383. doi: 10.1186/s13046-021-02193-1 (PMC8642961; doi:10.1186/s13046-021-02193-1)
Supplement: Supplementary file 2 — Additional file 2: Supplemental Table 1. sncRNAs in prostate cancer preclinical trials. [file 13046_2021_2193_MOESM2_ESM.docx]

**Supplemental Table 1. sncRNAs in prostate cancer preclinical trials**

| **Target Gene** | **Targeting effect** | **Therapeutic sncRNA** | **Reference** |
| --- | --- | --- | --- |
| **ADAM17** | Inhibition of cancer cell proliferation | siRNA | [1] |
| **AR** | Cell migration suppression and *in vivo* suppression of tumor growth and metastasis | siRNA | [2, 3] |
| **DNMT3** | Reduced proliferation, migration, and invasion | siRNA | [4] |
| **EGR-1** | Growth inhibition and stimulation of apoptosis | siRNA | [5] |
| **GRP78** | Reduced cellular migration and increased cell apoptosis | siRNA | [6] |
| **IGHG1** | Cell growth suppression, cell cycle arrest and apoptosis induction | siRNA | [7] |
| **JNK-1** | Induction of apoptotic signalling | siRNA | [8] |
| **Neu3** | Decreased cell invasion and migration, and in vivo reduction of bone metastases | siRNA | [9] |
| **RPL19** | Reduced xenografted tumor proliferation | siRNA | [10] |
| **SMAD7** | TGF-β signalling inhibitor | miR-21 | [11] |
| **USP9X** | Inhibition of tumor cell invasion | miR-1; miR-16 | [11] |
| **VEGF** | Inhibition of angiogenesis, cancerous cell proliferation and apoptosis induction | siRNA | [12] |

**References**

1. Lin F, Lin P, Liu X, Li D, Liu ZJ, Zou HF, Jiang Y, Zhao XF, Feng JL, Yu XG: **[Inhibitory effect of siRNA targeting ADAM17 on the proliferation of prostate cancer PC-3 cells].** *Zhonghua Nan Ke Xue* 2012, **18:**687-691.

2. Izumi K, Fang LY, Mizokami A, Namiki M, Li L, Lin WJ, Chang C: **Targeting the androgen receptor with siRNA promotes prostate cancer metastasis through enhanced macrophage recruitment via CCL2/CCR2-induced STAT3 activation.** *EMBO Mol Med* 2013, **5:**1383-1401.

3. Kim SS, Cho HJ, Kang JY, Kang HK, Yoo TK: **Inhibition of androgen receptor expression with small interfering RNA enhances cancer cell apoptosis by suppressing survival factors in androgen insensitive, late stage LNCaP cells.** *ScientificWorldJournal* 2013, **2013:**519397.

4. Du YF, Liang L, Shi Y, Long QZ, Zeng J, Wang XY, He DL: **Multi-target siRNA based on DNMT3A/B homologous conserved region influences cell cycle and apoptosis of human prostate cancer cell line TSU-PR1.** *Genet Mol Biol* 2012, **35:**164-171.

5. Parra E, Ferreira J, Saenz L: **Inhibition of Egr-1 by siRNA in prostate carcinoma cell lines is associated with decreased expression of AP-1 and NF-κB.** *Int J Mol Med* 2011, **28:**847-853.

6. Lu T, Yang W, Wang Z, Hu Z, Zeng X, Yang C, Wang Y, Zhang Y, Li F, Liu Z, et al: **Knockdown of glucose-regulated protein 78/binding immunoglobulin heavy chain protein expression by asymmetric small interfering RNA induces apoptosis in prostate cancer cells and attenuates migratory capability.** *Mol Med Rep* 2015, **11:**249-256.

7. Pan B, Zheng S, Liu C, Xu Y: **Suppression of IGHG1 gene expression by siRNA leads to growth inhibition and apoptosis induction in human prostate cancer cell.** *Mol Biol Rep* 2013, **40:**27-33.

8. Parra E: **Inhibition of JNK-1 by small interfering RNA induces apoptotic signaling in PC-3 prostate cancer cells.** *Int J Mol Med* 2012, **30:**923-930.

9. Li X, Zhang L, Shao Y, Liang Z, Shao C, Wang B, Guo B, Li N, Zhao X, Li Y, Xu D: **Effects of a human plasma membrane-associated sialidase siRNA on prostate cancer invasion.** *Biochem Biophys Res Commun* 2011, **416:**270-276.

10. Bee A, Brewer D, Beesley C, Dodson A, Forootan S, Dickinson T, Gerard P, Lane B, Yao S, Cooper CS, et al: **siRNA knockdown of ribosomal protein gene RPL19 abrogates the aggressive phenotype of human prostate cancer.** *PLoS One* 2011, **6:**e22672.

11. Bonci D, Coppola V, Patrizii M, Addario A, Cannistraci A, Francescangeli F, Pecci R, Muto G, Collura D, Bedini R, et al: **A microRNA code for prostate cancer metastasis.** *Oncogene* 2016, **35:**1180-1192.

12. Deezagi A, Ansari-Majd S, Vaseli-Hagh N: **Induced apoptosis in human prostate cancer cells by blocking of vascular endothelial growth factor by siRNA.** *Clin Transl Oncol* 2012, **14:**791-799.
